# Survival of patients with KRAS wild-type metastatic colorectal cancer is identical after sequential treatment with cetuximab and bevacizumab regardless of the sequence – A retrospective single-center study

**DOI:** 10.1093/gastro/gov051

**Published:** 2015-10-13

**Authors:** Yanhong Deng, Yue Cai, Jiayu Lin, Ling Jiang, Huabin Hu

**Affiliations:** Department of Medical Oncology, Sixth Affiliated Hospital of Sun Yat-sen University, Guangzhou, Guangdong, China

**Keywords:** metastatic colorectal cancer, sequential treatment, bevacizumab, cetuximab

## Abstract

**Objective: **To investigate the overall survival of patients with KRAS wild-type metastatic colorectal cancer (mCRC) after sequentially receiving both bevacizumab and cetuximab during the course of treatment.

**Methods: **Twenty-six mCRC patients who received both bevacizumab and cetuximab at the Sun Yat-sen University Gastrointestinal Hospital were retrospectively analyzed. Group A (n = 8) comprised patients who received bevacizumab first, and group B (n = 18) comprised those who received cetuximab first. The objective response rate, progression-free survival, and overall survival were compared.

**Results:** Baseline characteristics between the two groups were statistically similar. The objective response in groups A and B patients was 62.5% and 66.6%, respectively (*P* = 0.132). The median progression-free survival for groups A and B patients was 13 and 10 months, respectively (*P* = 0.798). The median overall survival for the entire cohort was 42 months, 44 months for group A and 39 months for group p B (*P* = 0.862) patients, respectively. Patients aged <40 years had worse survival than those aged ≥40 years (22 *vs* 44 months; *P* = 0.029). Patients with synchronous metastasis had worse survival than those with metachronous metastasis (unreached and 36 months, respectively). In multivariate analyses, synchronous metastasis and age remained statistically significant. The hazard ratio for synchronous metastasis was 4.548, and the HR for patients aged ≥40 years was 0.237.

**Conclusion:** A longer median survival time was observed in patients regardless of the targeted therapy sequence, which warrants further investigation.

## Introduction

Bevacizumab and cetuximab are two monoclonal antibodies that target vascular endothelial growth factor and epidermal growth factor receptor, respectively [1]. Although they have different mechanisms of action, both drugs are routinely used in combination with first-line chemotherapy for patients with metastatic colorectal cancer (mCRC) with KRAS wild-type exon 2 tumors [2,3]. With these two antibodies, as well as three traditional cytotoxic drugs including fluorouracil, oxaliplatin, and irinotecan, the survival of patients with KRAS wild-type exon 2 mCRC has greatly improved, approaching 30 months [4,5].

Extensive studies in phase II and III trials have been conducted to study the effects of starting treatment with either bevacizumab or cetuximab as both first- and second-line therapies. Two phase III head-to-head comparisons of bevacizumab and cetuximab trials attempted to assess the effects but did not reach the same conclusions [2,5]. A FIRE-3 trial was conducted by Heinemann *et al*.** that compared folinic acid, fluorouracil and irinotecan-based (FOLFIRI) therapy with cetuximab or bevacizumab in KRAS exon 2 wild-type patients [4]. Although the proportion of patients who showed an objective response did not exhibit significantly different results between the FOLFIRI-cetuximab and FOLFIRI-bevacizumab groups, a longer overall survival was observed in the FOLFIRI-cetuximab group [2]. However the suggestion that cetuximab and bevacizumab are interchangeable agents in the first-line setting of mCRC is supported by results of the CALGB/SWOG 80405 trial [5]. These trials triggered a number of debates on what caused the different results and how to guide clinical practice. Although these were high-quality trials, the drawback of both is that second-line treatment, which plays an increasingly significant role in prolonging overall survival of patients with mCRC, was not warranted.

This study was undertaken to investigate the overall survival time for patients who are sequentially administered with both bevacizumab and cetuximab.

## Patients and methods

### Patient population

Patients with KRAS exon 2 wild-type mCRC who were treated with palliative aim and who received both cetuximab and bevacizumab sequentially were selected from the database of Sun Yat-sen University Gastrointestinal Hospital from January 2009 to April 2013 for this retrospective study. The study was approved by the Medical Ethics Board of Gastrointestinal Hospital, Sun Yat-sen University. The baseline clinicopathological factors were recorded including sex, age, cancer antigen-199 (CA199) level, hemoglobin level, carcinoembryonic antigen (CEA) level and lactate dehydrogenase level.

### Chemotherapy regimens

The doublet regimen consisted of fluorouracil (5-FU) (including capecitabine) combined with either oxaliplatin or irinotecan and was the backbone chemotherapy. Together with this therapy, 5.0 mg/kg bevacizumab every two weeks or 7.5 mg/kg every three weeks was administrated. The dose for cetuximab was 500 mg/m^2^ every two weeks or 400 mg/m^2^ for the first week and 250 mg/m^2^ each following week. Patients using bevacizumab before cetuximab were placed in group A; those using cetuximab before bevacizumab were placed in group B.

### KRAS mutational analysis

The primers for the amplification and Sanger dideoxy chain termination sequencing of KRAS gene were forward: 5’- GTCCTG CACCAGTAATATGC-3’ and reverse: 5’-ATGTTCTAATATAGTCACATTTTC-3’ for codon 12 and 13. Polymerase chain reaction (PCR) was performed using 100 ng genomic DNA as the template. Each mixture contained 10 pmol of each primer. The reactions were performed in a total volume of 31.45 µl .The amplification reactions were as follows: an initial denaturing cycle of 95°C for 5 min; 45 cycles of 94°C for 25 s, 58°C for 25 s, 72°C for 25 s; and a final extension cycle at 72°C for 10 min. The PCR products were then purified and subjected to direct sequencing using the automatic sequencer (ABI-3730 Genetic Analysis, Applied Biosystems).

### Clinical evaluation and follow-up

All patients received a chest, abdomen, and pelvic CT scan or PET/CT scan to gather baseline data and were reevaluated at 1.5–3 month intervals according to the clinical manifestations and physicians’ decisions. In addition to the above tests, evaluations consisted of pertinent medical history, physical examination, blood cell counts, blood chemistry assessment, CEA and CA190. RECIST 1.1 criteria were used to define the efficacy as complete response (CR), partial response (PR), stable disease (SD) and progression of disease (PD). Progression-free survival (PFS) was defined as the time from the treatment date to the date of disease progression or death, whichever happened first. Overall survival (OS) was defined as the time from the date of diagnosis with mCRC to death.

### Statistical analyses

Statistical analyses were performed using SPSS 17.0. Categorical data were analyzed by using Fisher’s exact tests. Continuous data were analyzed by using the Student* t* test. Survival curves were generated according to the method of Kaplan–Meier, and univariate survival distributions were compared using the log-rank test. Hazard ratios (HRs) and 95% confidence intervals (CIs) for univariate and multivariate models were computed using Cox proportional-hazards regression. All reported *P* values are two sided, and *P* values < 0.05 are considered statistically significant.

## Results

### Patient characteristics

Twenty-six patients, comprising 9 females and 18 males, were recruited. The ages ranged from 26 to 74 years with a median age of 48 years. Eleven patients had more than two organs involved in the disease at the initial diagnosis. Eight patients started therapy with bevacizumab (group A), and 18 began with cetuximab (group B). The baseline characteristics were not significantly different between the two groups in terms of age, sex, differentiation, involved organs, synchronous metastasis and CEA level (Table 1).

**Table 1.** Patient characteristics

	Group A	Group B	*P* value
Age (years)	49.5 + 15.4	47.7 + 14.2	0.779
Female sex	5 (62.5%)	4 (22.2%)	0.078
Involved organs >1	5 (62.5%)	6 (33.3)	0.105
Synchronous metastasis	7 (87.5%)	6 (66.7%)	0.375
Differentiation			0.802
High	3 (37.5%)	5 (27.8%)	
Moderate	4 (50.0%)	9 (50.0%)	
Poor	1 (12.5%)	4 (22.2%)	
First-line chemotherapy regimens			0.653
Oxaliplatin based	7 (87.5%)	12 (66.7%)	
Irinotecan based	1 (12.5%)	6 (33.3%)	
Carcinoembryonic antigen (ng/ml)	67.1 + 85.0	122.9 + 244.6	0.400
Lactate dehydrogenase (U/L)	206.4 + 36.3	276.8 + 13.8	0.314
Hemoglobin (g/L)	119.5 + 29.4	121.4 + 13.8	0.869
Platelet (×10^9^)	287.7 + 137.8	2114.3 + 98.9	0.206
CA199 (U/L)	110.6 + 207.8	108.2 + 260.6	0.980

Data expressed as mean ± standard deviation or number (%). *P* values were derived using Fisher exact tests and Student *t* tests for categorical and continuous variables, respectively.

### Objective response

CR was seen in one patient (12.5%), and PR was seen in four patients (50%) in group A. There was no CR in group B, but PR was seen in 12 of the 18 (66.6%) patients. There was no significant difference in terms of objective response (*P* = 0.132). No PD was recorded in either group.

### Progression-free survival

**Figure 1.**
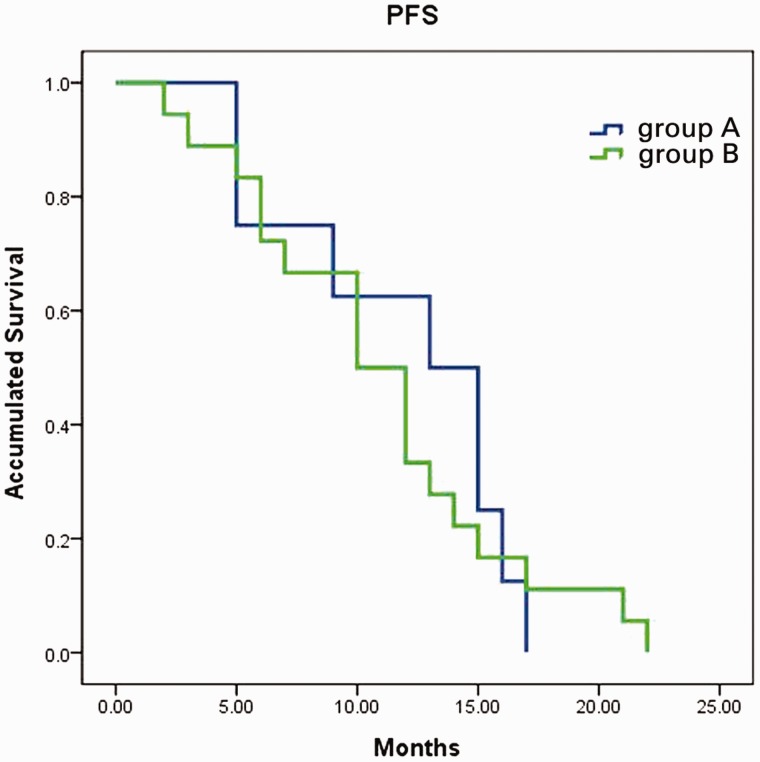
Progression-free survival curves of group A and group B patients. Progression-free survival was identical for patients who received bevacizumab (group A) first or cetuximab first (group B) (13 *vs* 10 months, *P* = 0.798).

The median follow-up time for the cohort was 31 months. All patients experienced PFS, and there was no difference in PFS between the two groups. The median PFS for group A and group B patients was 13.0 (95% CI: 7.5–18.5) and 10.0 (95% CI: 6.5–13.5), respectively (*P* = 0.798; Figure 1).

### Overall survival

**Figure 2.**
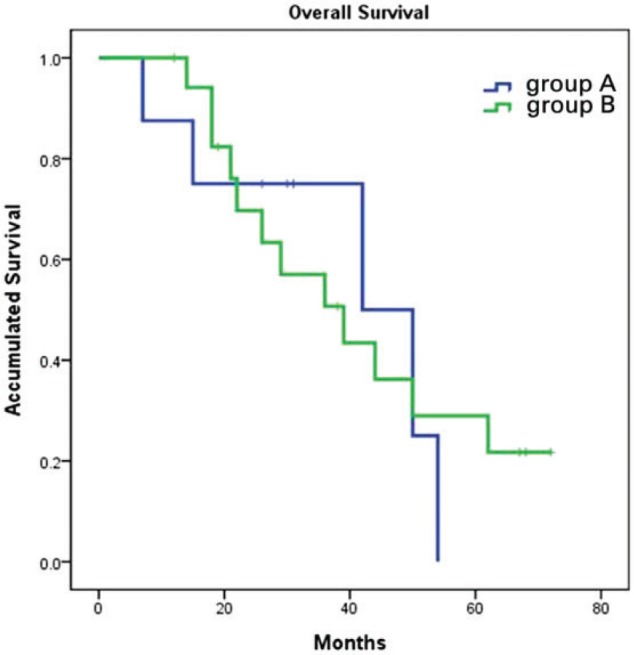
Overall survival curves of group A and group B patients. Overall survival was identical for patients who received bevacizumab (group A) first or cetuximab first (group B) (44.0 *vs* 39.0 months, *P* = 0.862).

**Figure 3.**
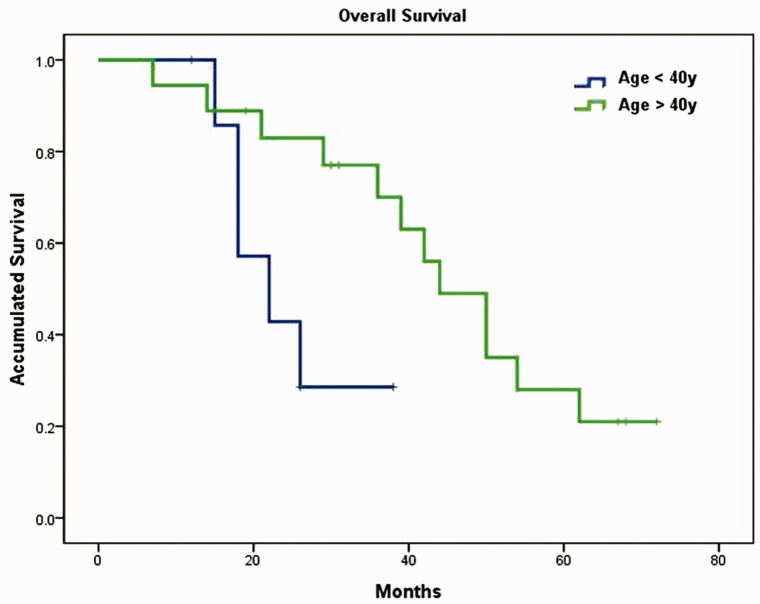
Overall survival differences between young and old patients. Patients aged ≥40 years had significantly better survival than those aged <40 years (44 *vs* 22 months, *P* = 0.029).

**Figure 4.**
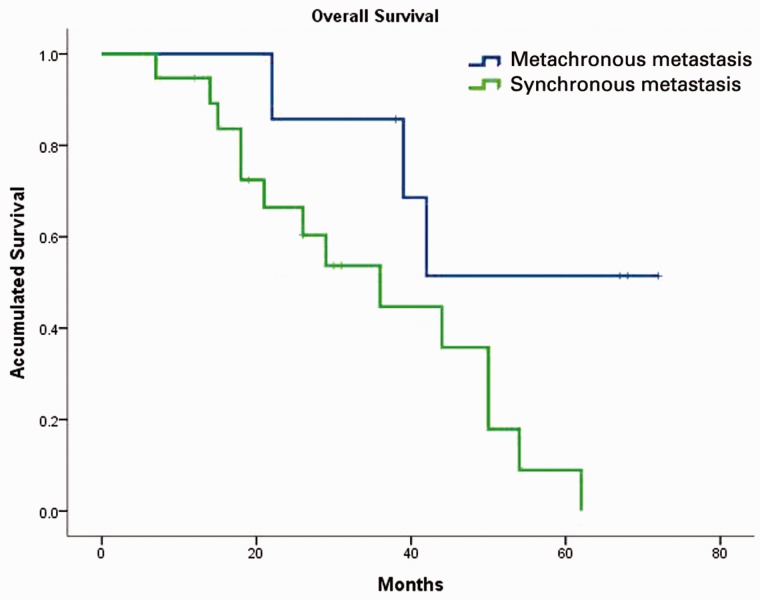
Overall survival difference between synchronous and metachronous patients. Patients with synchronous metastasis had lower survival rates than those with metachronous metastasis (36 months *vs* unreached, *P* = 0.038).

Median OS for the entire cohort was 42 months (95% CI: 32–51.99) with 44 months (95% CI: 10.7–73.3) in group A and 39 months (95% CI: 20.8–57.2) in group B. There was no significant difference between the two groups (*P* = 0.862; Figure 2). In contrast, subgroup analysis showed that patients aged ≥40 years had significantly better OS than those aged <40 years, median OS was 44 months (95% CI: 34.4–53.6) and 22 months (95% CI: 11.7–32.3), respectively (*P* = 0.029; Figure 3). Patients with synchronous metastasis (median OS: 36 months, 95% CI: 19.7–52.3) had worse survival than those with metachronous metastasis (median OS was not reached, *P* = 0.038; Figure 4). When age, differentiation, CEA level, involved organs and synchronous or non-synchronous metastasis were analyzed by multivariate analyses, synchronous metastasis and age remained significant. The HR for synchronous metastasis was 4.548 (95% CI 1:.147–18.034), and the HR for age ≥40 years was 0.237 (95% CI: 0.060–0.928).

## Discussion

Combinations of chemotherapy regimens and monoclonal antibodies have demonstrated their efficacy for improving the clinical outcomes of patients with mCRC and resulted in achieving an overall survival time > 30 months [4,5]. Biologic drugs combined with doublet chemotherapy regimens have become the first choice for most patients; however, for KRAS wild-type patients, therapy beginning with either cetuximab or bevacizumab has been extensively studied in phase II and III trials, both as first- and second-line therapies. Subject to the knowledge of the KRAS status of the tumor, the targeted agent with which to combine first-line chemotherapy should still be selected based on patient and physician assessment of the risk:benefit ratio of cetuximab compared with that of bevacizumab in individual patients.

The status of the debate about which drug is better remains the same as that 10 years ago, when both oxaliplatin and irinotecan were approved for mCRC first-line treatment [6]. The battle between oxaliplatin and irinotecan stopped after the GERCOR study V308 [7] showed identical results of both treatments. Shortly thereafter, Grothey *et al* built a model that illustrated that a strategy of making all active agents available to patients with advanced mCRC appears to be more important than the use of irinotecan- or oxaliplatin-based combination therapy upfront [8]. From the results of our current study, we believe that the strategy of making all active agents available is still applicable and very important in the five-drug era. When patients were exposed to five active drugs, regardless of which antibody (bevacizumab or cetuximab) was used first, the median overall survival reached 42 months (95% CI: 32–51.99). Among the reported clinical trials, only first-line treatment was the focus; no second-line treatment was defined, although it is very important for the overall survival of patients with mCRC. The results of the STRATEGIC-1 study, conducted by the GERCOR group in which two strategies rather than two drugs are compared, are not yet available.

Our results also indicate that metachronous metastasis patients have a significantly better survival rate than synchronous metastasis patients. The median overall survival for metachronous metastasis patients was not reached after the latest follow up. The result is consistent with those of other reports [9], but whether it is because of prognostic differences or differences in chemosensitivity warrants further investigation. Another indication is the difference in the median overall survival rate between patients younger than aged 40 years and those aged 40 years and older. The older patients had a better overall survival of 48 months, whereas the younger patients had a median overall survival of only 22 months. It may be due to the fact that tumor cells arising from younger patients have more aggressive biological features; however, studies demonstrated controversial results [10,11]. Fu *et al*.** found that patients younger than aged 35 years who were diagnosed with CRC had a worse prognosis because of a higher proportion of advanced-stage tumors [11]. When stage-to-stage analysis was performed, it was found that young adult CRC patients had a worse outcome only if they had stage IV tumors [11]. Nevertheless, our study demonstrated an extremely large difference between the two groups (22 *vs* 48 months), which emphasizes that younger patients need more effective treatments.

Our current study is limited by being a retrospective analysis with a small sample size. However, the conclusions from this study warrant further investigation.
